# Editorial: The use of ketogenic diet therapy in the era of individualized therapy

**DOI:** 10.3389/fnut.2023.1272170

**Published:** 2023-09-19

**Authors:** Aycan Ünalp, Bülent Ünay, Ebru Arhan

**Affiliations:** ^1^Department of Pediatric Neurology, Izmir Faculty of Medicine, University of Health Sciences (Turkey), Izmir, Türkiye; ^2^Dr. Behçet Uz Children's Hospital, University of Health Sciences, Izmir, Türkiye; ^3^Department of Pediatric Neurology, Gulhane Faculty of Medicine, University of Health Sciences (Turkey), Ankara, Türkiye; ^4^Department of Pediatric Neurology, Gazi University Hospital, Ankara, Türkiye

**Keywords:** individualized, personalized, ketogenic diet, therapy, precision

The term individualized medicine refers to truly personalized treatment that tries to treat each patient according to their individual biology.

“Personalized” medicine is a model of N-of-1, in which each patient is considered the only patient treated. “Precision” medicine, on the other hand, resembles the 1-in-N model, allowing a more traditional Western medicine approach to conducting research on groups and sub¬groups and treating the patient's specific subgroup (1, 2). Thus, individualized medicine can go one step further and be considered as personalized medicine.

This Research Topic aimed to assess ketogenic diet usage, acceptability, and efficacy of individualized medicine.

In this special e-collection, we tried to collect and combine the articles that will help us understand the effects and mechanisms of ketogenic diet therapy in special patients and/or patient groups in which they are used individually.

Ketogenic dietary therapies are well-established, safe, non-pharmacologic treatments used for children and adults with drug-resistant epilepsy and other neurological disorders (3). Ketone body levels are recognized as helpful to check compliance to the ketogenic diet therapy and to attempt titration of the diet according to individualized needs. Possible variations in glycemia and keton bodys blood levels according to the menstrual cycle have not been systematically assessed yet, but this time window deserves special attention because of hormonal and metabolic-related changes. Pasca et al. aimed at searching for subtle changes in the ketone body's blood level during the menstrual cycle in female patients. A significant increase in glucose blood levels during menstruation was found in the entire cohort. As far as the keton bodys blood levels, an inversely proportional trend compared to glycemia was noted.

The classic ketogenic diet is an isocaloric, high-fat, low-carbohydrate diet that induces the production of keton bodys. High consumption of dietary fatty acids, particularly long-chain saturated fatty acids, could impair nutritional status and increase cardiovascular risk. De Amicis et al. evaluated the long-term effects of a 5-year classic ketogenic diet on body composition, resting energy expenditure, and biochemical parameters in children affected by Glucose Transporter 1 Deficiency Syndrome (GLUT1DS). Long-term adherence to the classic ketogenic diet showed a good safety profile on anthropometric measurements, body composition, resting energy expenditure, and biochemical parameters. Ketogenicdietary treatments are to date the gold-standard treatment for GLUT1DS. Administration of ketogenic diet therapy is generally per os; however, in some conditions including the acute gastro-enteric post-surgical setting, short-term parenteral administration might be needed. De Giorgis et al. reported the first pediatric patient with GLUT1DS in chronic treatment with ketogenic diet therapy efficiently treated with exclusive parenteral nutrition for 5 days. This case reports on real-world management and the ideal recommendations for parenteral-ketogenic diet therapy in an acute surgical setting.

COVID-19 is associated with subclinical myocardial injury. Exogenous ketone esters acutely improve left myocardial function in healthy participants and patients with heart failure, but the effects have not been investigated in participants previously hospitalized for COVID-19. Wodschow et al. performed a randomized placebo-controlled double-blind crossover study. They concluded that in patients previously hospitalized with COVID-19, a single oral dose of ketone ester had no effect on left ventricular ejection fraction (LVEF), cardiac output or blood oxygen saturation, but increased global longitudinal strain (GLS) acutely.

The ketogenic diet, as a dietary intervention, has gained importance in the treatment of solid organ structural remodeling, but its role in renal fibrosis has not been explored. Qiu et al. demonstrated that a ketogenic diet significantly enhanced serum β-OHB levels in mice. Histological analysis revealed that the ketogenic diet alleviated structural destruction and fibrosis in obstructed kidneys and reduced the expression of the fibrosis protein markers α-SMA, Col1a1, and Col3a1. Their results highlight that the ketogenic diet attenuates unilateral ureteral obstruction (UUO)-induced renal fibrosis by enhancing fatty acid oxidation (FAO) via the free fatty acid receptor 3 (FFAR3)-dependent pathway, which provides a promising dietary therapy for renal fibrosis.

High carbohydrate, low fat (HCLF) diets have been the predominant nutrition strategy for athletic performance. Highly trained competitive middle-aged athletes underwent two 31-day isocaloric diets (HCLF or LCHF) in a randomized, counterbalanced, and crossover design while controlling calories and training load. These results: (i) challenge whether higher carbohydrate intake is superior for athletic performance, even during shorter-duration, higher-intensity exercise; (ii) lower carbohydrate intake may be a therapeutic strategy to independently improve glycemic control, particularly in those at risk for diabetes; (iii) unique relationship between continuous glycemic parameters and systemic metabolism (Prins et al.).

Sub-acute sclerosing panencephalitis (SSPE) is a chronic, progressive neurodegenerative disorder, commonly seen in measles-endemic countries leading to progressive neuronal loss and death. Currently, there is no proven cure for this devastating disease. Ibrahim and Farooq  evaluated 12 children whoes low glycemic index diet (LGIT) was started with a confirmed diagnosis of SSPE. Seven (58.3%) children showed a >50% reduction in myoclonic jerks with three (25%) having a 100% reduction. LGIT may play an effective role in the management of SSPE.

Glioblastoma Multiforme is an aggressive brain cancer affecting children and adults frequently resulting in a short life expectancy. The analysis revealed several limitations of the ketogenic diet as an intervention. The effectiveness is more robust in mice than in human studies. Furthermore, tolerability is marginally supported in human studies requiring more reproducible research to validate that the intervention is manageable and effective in patients with glioblastoma (Clontz). Mast cell tumors (MCT) are common neoplasms in dogs and are similar to most other malignant cancers in requiring glucose for growth, regardless of histological grade. Ketogenic metabolic therapy (KMT) is emerging as a non-toxic nutritional intervention for cancer management in animals and humans alike. Seyfried et al. reported the case of a 7 years-old Pit Bull terrier that presented in 2011 with a cutaneous mast cell tumor under the right nostril. The resolution of the tumor in this canine patient could have been due to the diet-induced energy stress and the restriction of glucose-driven aerobic fermentation that is essential for the growth of most malignant tumors.

In summary this Research Topic support that ketogenic diet therapies are effective and safe treatment for many diseases and is promising as an individualized treatment.

## Author contributions

AÜ: Writing—review and editing. BÜ: Writing—review and editing. EA: Writing—review and editing.
